# NEWS 1

**DOI:** 10.7189/jogh.07.010201

**Published:** 2017-06

## Regions

### Africa

➢ New regional health centers, the Africa Centres for Disease Control and Prevention, will be set up across Africa to tackle health threats. These Centres will partner with the US, EU and China, with technical advice from the WHO. The announcement was made at February’s summit of the African Union (AU) in Addis Ababa, Ethiopia, alongside a commitment to make immunisation available across the continent by 2020. Currently, 1 in 5 children in Africa are without access to basic immunisation, while conflict states have experienced a resurgence in diseases like polio. Although immunisation has increased in Africa over the past 15 years, Africa is falling behind on vaccination targets, hampered by hot climates, unreliable electricity, and supply shortages. Fewer than 20 countries fund more than 50% of their immunisation programmes, but as more countries approach middle–income status they will become ineligible for GAVI support for their immunisation programmes. Dr Ngozi Okonjo–Iweala, the chair of GAVI, commended the AU’s leadership, and called for sustainable immunisation financing. “Immunisation is one of the smartest investments a country can make in its future. When our children are healthy, our families, communities and countries thrive,” said Yifru Berhan Mitke, Ethiopia’s health minister. (The Guardian, 1 February 2017)

➢ In South Africa, miner Bongani Nkala and 55 others have brought a class action against 32 of the world’s largest gold mining companies, seeking compensation for up to 500 000 miners who may have contracted silicosis and tuberculosis while working underground. One estimate of the total compensation due is US$ 3.25 billion. Silicosis is an incurable disease caused by inhaling silica particles, and is also a strong risk factor for tuberculosis. In 2016, South Africa’s Supreme Court ruled that their application – the largest class action in South Africa’s history, and the first concerning an employer exposing workers to harmful quantities of dust underground – could proceed, although the 32 companies are seeking leave to apply and hence delay any compensation payments. The companies are also petitioning against the transferability of compensation from deceased miners to their spouses and families. There are concerns that the appeals process could continue for another 18 months – when many of the claimants are seriously ill and elderly. For the past 100 years, the problem of miners suffering from lung diseases went ignored and uncompensated, and under apartheid was systematically under–reported. Moreover, in order to qualify for compensation, a miner’s lung function must be impaired by more than 10%, leading to many cases being rejected – and death certificates recording tuberculosis often missed the underlying cause of silicosis. (Daily Maverick, 2 March 2017)

➢ By early April, 4637 cases of cerebrospinal meningitis were recorded across Nigeria, killing at least 489 people. This announcement was made at an emergency meeting of northern state governors and traditional rulers, by the country’s health minister, Mr Isaac Adewole. Mr Adewole confirmed that so far 823 000 doses of the conjugated Type C vaccine had been delivered to the Federal Government by the UK government, and UNICEF had delivered over 1 million Type A vaccines. He also confirmed that the Federal Government would spend US$ 1 billion to procure vaccines to eradicate meningitis in the 5 most affected states in North Nigeria. He highlighted how the federal government had collaborated with state governments and aid organisations to tackle the outbreak across 26 states, and called for traditional rulers to spread the message on prevention, plus for more investment in health systems at the state level to tackle health challenges. He also called for corruption to be tackled to ensure that funds are properly utilized. (Premium Times, 11 April 2017)

➢ As countries in Africa urbanise, disease patterns change and more people can afford health care or health insurance, bringing opportunities for private companies. Africa’s disease pattern is moving from infectious diseases toward non–communicable diseases, and the WHO estimates that 1 million people will die of cancer each year in Africa by 2030. Moreover, the IFC (the private–sector arm of the World Bank) estimated that US$ 25–30 billion of investment will be required in health care infrastructure up to 2022. These trends are an opportunity for private companies, as African health care systems are generally developing from a low base. GE Healthcare Africa has adapted its business model toward developing innovative financing and training practitioners, and by public–private partnerships to supply equipment. Private equity firms are also investing in African health care companies, eg, Investment Funds for Health in Africa has invested in 12 companies covering care delivery, medical supplies and distribution, plus hospitals and private clinics. In addition, the Egyptian conglomerate the Mansour Group, are considering expanding into health care. “Nigeria has almost 200 million inhabitants and they have four MRI machines. It doesn’t take rocket science to see the opportunity,” says Mohamed Mansour, group chairman. (Financial Times, 10 May 2017)

➢ Biovac, a South African company, and PATH [a global health, non–profit organization] will develop a new vaccine against Group B Streptococcus (GBS) – one of the leading causes of infections in infants. This partnership with PATH, funded by the Bill and Melinda Gates Foundation, makes Biovac one of only 3 companies working on a conjugate vaccine against streptococcus – and the only one in a developing country. South Africa has one of the world’s highest incidences of streptococcus infections – up to 3 in every 1000 live births – and deaths can occur in up to 4% of infections, and survivors can face life–long impairments, such as blindness and developmental impairments. Streptococcus can affect all age groups, but newborns are particularly vulnerable because of their immature immune systems. Infections can be prevented by antibiotic treatment given to pregnant women, but this is not available is most resource–limited countries, and is less effective against late–onset GBS. According to Biovac’s CEO, Dr Morena ‘Makhoana, this is an opportunity for Biovac to not only be a manufacturer but “a true developer of novel vaccines aimed at African and developing–world diseases.” (Daily Trust, 19 May 2017)

### Asia

➢ According to researchers from Imperial College London, South Korea is set to have the longest life expectancy by 2030, overtaking the current holder, Japan. The average lifespan of Japanese women is forecast to be overtaken by South Korea and France, while Japanese men will drop from 4th to 11th place in the global rankings. South Korean women will be the first to have an average life expectancy of more than 90 years, and men will have an average life expectancy of 84 years. The study authors believe that these developments are mainly due to investments in universal health care, education, childhood nutrition and the rapid scale–up of new medical technologies, underpinned by lower body–mass indices and blood pressure. This highlights South Korea’s remarkable transformation from an impoverished, war–torn country 60 years ago into today’s modern, technologically–advanced powerhouse. However, a note of caution in interpreting these figures was sounded by Prof Park Eun–cheol, who highlighted that the country’s low fertility rate means a very low incidence of child mortality, which affects life expectancy calculations. In addition, nearly 50% of South Koreans aged 65 years or above live in poverty – the highest rate in the OECD. (Financial Times, 22 February 2017)

➢ After no indigenous cases of malaria for the past 3 years, Sri Lanka was declared malaria–free by the World Health Organization – bringing the number of cases from 264 549 in 1999 to 0 in November 2012. Sri Lanka joins 17 countries that have eliminated malaria, and India plans to join this group by 2030 – in India, 1.06 million people were infected by malaria in 2015, with 242 confirmed deaths. Sri Lanka deployed various tools in its fight against malaria, such as: web–based surveillance, including testing all fever cases for malaria, and monitoring travelers from countries with malaria–transmission; a 24–hour telephone helpline to notify, track and treat malaria patients in isolation to halt the spread of infection; changing emphasis from mosquito–control to parasite control; strengthening public health systems, improved sanitation and transport infrastructure to make health care more accessible; and working in partnership with other stakeholders on disease surveillance, and social, technical and financial support for malaria eradication. (Hindustan Times, 5 May 2017)

➢ Bangladesh has approved construction of a giant coal–fired power station, and the environmental campaigning organization Greenpeace warns that it could worsen air pollution for millions of people, and cause the early deaths of 6000 people over its lifetime. The plant will be erected on the edge of the world’s largest mangrove forest, also threatening the area’s fragile ecosystem, as the forest is a barrier against storm surges and cyclones – and violent weather patterns have killed thousands of people in the area. Greenpeace also warns that the plant could render fish unsafe to eat for millions across the Bay of Bengal due to mercury deposits, and devastate the aquatic food chain, as it is projected to discharge nearly 125 000m^3^ of waste–water into nearby water catchments. Mr Sheikh Hasina, Bangladesh’s Prime Minister, defended the project and rejected concerns as being politically motivated. However, it has galvanised street protests in Bangladesh, with campaigners calling for the plan to be scrapped or relocated. (Arab News, 5 May 2017)

➢ Laos held its first National Conference on Family Planning, as part of its strategy to move away from Least Developed Country Status by 2020. The conference’s theme was “investing in family planning for economic prosperity”, and was supported by the UN Population Fund. Despite Laos’s progress in reducing maternal mortality and improving access to contraception, its rate of maternal deaths remains high (206 per 100 000 births), with correspondingly high levels of teenage pregnancies. Contraceptive use stands at 50%, and 20% of all family planning needs are unmet – with rates much higher in some remote areas. The Laotian government plans to spend US$ 15 million over the next 4 years, focusing on promoting long–acting reversible contraception, increasing the number of midwives, improving capacity at health centers, and campaigns aimed at young people. According to the UN Population Fund’s representative, Frederika Meijer, the conference represented a shift from viewing contraception as merely a way of limiting population, toward viewing it as a way of saving lives, and improving quality of life, economic prosperity and development. (News Deeply, 19 May 2017)

➢ In the first cross–border civilian exchanges since North Korea’s 4th nuclear test in 2016, South Korea will allow a civic group to contact North Korea to help fight malaria. The announcement signaled that South Korea’s new government may try to re–start civilian aid and exchange programmes as part of improving tense relationships between the countries. The civic group will have email contact with North Korea to offer insecticides, diagnostic kits, mosquito repellants and nets – the first time the group had sent such supplies since 2011. According to the World Health Organization, North Korea had 7010 malaria cases in 2015, compared to 21 850 in 2012, partly due to international anti–malaria aid programmes. Malaria in North Korea’s southern–most regions is also a risk for South Korea, as mosquitoes can readily cross the heavily–fortified border. (ABC News, 26 May 2017)

### Australia and Western Pacific

➢ Australia was the first country to introduce mandatory plain packaging for tobacco products, and plans to increase tobacco taxes until a packet of cigarettes costs US$ 40. Some Australian states have also introduced stringent anti–smoking measures, such as prohibiting smoking with 10m of a playground, or 4m from the entrance to a public building, and banning smoking on many beaches. This measures are enforced by heavy fines in many places. These measures, combined with anti–smoking campaigns, have caused smoking rates to fall by almost 50% since 1980 – in Australia, the smoking rate is 13% compared to a global average of 20%, and there has been a 23% decrease in hospital admissions for smoking–related illnesses. The Australian government has committed to reducing the percentage of adult smokers to 10%, and Tasmania is discussing a bill to outlaw smoking for those born after 2000. A “culture of shame” around smoking is beginning to emerge in Australia, which also acts as a deterrent. However, smoking is mainly taken up by the poorest in Australia society, so the trend to stigmatise smoking may add a burden of shame to those who are already marginalised. (BBC, 30 January 2017)

➢ Australia’s mosquito–borne Ross River virus (RRV) – the country’s most common mosquito–borne disease– was reported in higher numbers in 2016 due to unusually wet weather – and could become a global epidemic. Previously, RRV was believed to be confined to Australia and Papua New Guinea, but there is evidence that it may have spread overseas, with some cases being reported in the South Pacific. This was uncovered when travelers to the South Pacific were infected with RRV despite never visiting Australia or Papua New Guinea – and contrary to the earlier belief that RRV was only transmitted via marsupials, thus self–limiting itself to Australia and Papua New Guinea. This has caused concerns that the virus is self–sustaining, and could become global. RRV is not a deadly disease, but it can be difficult to diagnose as its symptoms are very general (including swollen joints, fever, fatigue and pain), and in 2016 more than 3500 Australians were infected. (Xinhua, 23 February 2017)

➢ According to a nationwide survey, 1–in–6 unvaccinated children in Australia have been denied treatment because their immunisations were not up–to–date. The findings came from the Melbourne Royal Children’s Hospital Child Health Poll, which surveyed nearly 2000 parents of 3500 children. The vast majority of the parents surveyed had their children fully immunised, with 6% were being selectively unvaccinated, and only 1% completely unvaccinated. Federal Health Minister Greg Hunt will refer the findings about care refusal to the Australian Health Practitioners’ Regulatory Agency for further investigation. The President of the Australian Medical Association, Michael Gannon, emphasized that refusing care to anyone, particularly children who cannot choose their immunisation status, was unethical. “No matter what reservations an individual doctor might have, it’s not ethical to deny care to an unvaccinated child,” he said, although he recognized that individual doctors may need to vary their practice depending on risks to other patients, eg, a child with measles could infect other children in the waiting room. (Sydney Morning Herald, 7 March 2017)

➢ The Federated States of Micronesia, the Marshall Islands and the Republic of Palau are covered by the Compact of Free Association (COFA) agreements, which puts them under the protection of the US Government. This entitles citizens of these countries to live and work in the USA and currently 6500 people from these nations live in Washington DC, but they currently do not qualify for Medicaid. These nations were the site of more than 60 nuclear tests carried out by the USA between 1946 and 1958, and these tests still adversely affect their citizens’ health today. However, a bill is being passed that would help Washington’s COFA citizens to pay for insurance under the state health insurance exchange, and would allocate US$ 3.9 million over the next three years to support it. To qualify, a COFA islander has to live in Washington, earn up to 133% of the federal poverty level, and be ineligible for any other state or federal benefits, including Medicaid. Washington State would be required to set up enrolment procedures at the state health insurance exchange, and carry out public outreach. (Seattle Globalist, 8 March 2017)

➢ There have been a few cases of newborn hemorrhagic disease, caused by a Vitamin K deficiency, due to parents refusing the routine post–birth Vitamin K injection for their babies. Up until the early 1970s, the disease killed 15 newborn babies each year in New South Wales, but the introduction of routine Vitamin K injections at birth virtually eliminated it. However, one baby died in 2013 and another in 2012, and pediatricians have reported regularly having to convince parents to get the Vitamin K shot. It appears that the home–birth and anti–vaccination movement are often involved in falsely persuading parents of the dangers of the Vitamin K injection. (The Mercury, 11 March 2017)

### China

➢ A variety of *E coli* bacteria in China has recently evolved resistance to colistin, a key antibiotic of last resort. The gene conferring resistance, mcr–1, was found in 1% of *E coli* bacteria and 1% of *Klebsiella pneumonia* bacteria, which can cause pneumonia, and blood and wound infections. The mcr–1 gene moves easily from host to host, and also across types, eg, *E. coli* and *Klebsiella*. In many countries, the use of colistin is reserved for human use, and in emergencies only. However, it is widely used in China for agricultural purposes, eg, fattening farm animals. China will ban the use of colistin in agriculture from 1 April, licensing it for human use only to deal with the spread of other superbugs in China. It will be required to deal with China’s problem of carbapenem–resistant *Enterbacteriaceae* – gut bacteria which are not susceptible to the key antibiotic group of carbapenems. There is a danger than resistance to colistin and carbapenem may converge – although it appears not yet to be a significant problem in China – and that standard medical procedures could become too dangerous to perform. (Scientific American, 27 January 2017)

➢ China’s plan to increase the number of doctors by nearly 40% over the next 5 years is threatened by medical graduates choosing other professions. China needs to rely more on general practitioners, or family doctors, to relieve the pressure placed on underfunded public hospitals from an aging population and the increasing burden of non–communicable diseases such as diabetes. Moreover, the State Council’s plan to increase average life expectancy also includes plans for more doctors, and China will need an estimated 140 000 additional obstetricians and midwives after it scrapped its one–child policy to allow couples to have two children. But it may be difficult to recruit doctors in sufficient numbers, as the total number of doctors rose by just 750 000 from 2005–2015, while China produced 4.7 million medical graduates. Low salaries are one factor behind this leakage, as the average doctor’s salary is US$ 720/month; another factor is overwork, as doctors will often see up to 12 patients per hour, and can be attacked by patients and their families who are frustrated over medical care. The shortage is particularly acute in the countryside, with a shortfall of 500 000 doctors, and in certain specialties such as pediatrics. (Financial Times, 19 January 2017)

➢ China has established its first big data research centre for children’s health, to improve the health of the country’s children by developing a more complete health system for children, focusing on disease prevention, diagnosis and personalised treatment. It was established by Wuhan University and a Beijing technology firm in central China’s Hubei province, and to date has collected information on more than 200 000 children in 70 hospitals across 7 provinces, expanding to 300 hospitals by 2017 and 1000 by 2020. The research centre plans to develop into a national cloud platform for children’s health information, offering standards on personalised medical care and clinical treatment. (Xinhua, 11 March 2017)

➢ China has already experienced remarkable progress in lifting people from poverty – 800 million people over the past 35 years, and the country’s government has pledged to effectively eradicate rural poverty by 2020, moving 45 million people above the rural poverty line of US$ 324 per year (ie, the average income of the poorest 5% of rural households, which compares to US$ 1128 for urban households). In recent years, the pace of poverty reduction in China has slowed, from 26 million people a year up to 2000, to 22 million a year from 2000–15. The government now has a target of lifting 10 million people from poverty each year. As the rate of poverty reduction has slowed, its costs have risen – in 2000, it cost approximately US$ 48 per year to lift a person out of poverty, and by 2010 this increased to US$ 150 per year. This illustrates how the people who benefit most from poverty reduction are generally those who are best equipped to do so – those who are left behind are generally harder to reach, and may lack access to roads, electricity and clean water. The cost of lifting those still below the rural poverty line out of poverty is more than US$ 200 a year. However, in 2016 the government exceed its annual target, as 12.4 million rural people left poverty, and its 2017 budget is 30% larger, meaning that at least US$ 1000 has been allocated for each person the government plans to lift out of poverty in 2017. (Project Syndicate, 28 March 2017)

➢ Yi Fuxian, a demographer working at the University of Wisconsin–Madison, has claimed that Chinese statisticians have overestimated the country’s population by 90 million, partly by inflating fertility rates, and that India may have overtaken China as the world’s most populous country. He estimates that China’s population is 1.29 billion, compared to the government’s estimate of 1.38 billion, while India’s population is 1.32 billion. This claim was disputed by other demographers, while still accepting the country’s very low–birth rate, counter–argue that the government’s figures are correct. India’s demographers also accept that China has a higher population, but predict that India will overtaken China by 2025. Wang Feng, a demographer at the University of California, dismissed Yi Fuxian’s claims; although he believes that the government may have slightly overestimated the country’s population, the error is much less than 90 million people. “That is two Spains. It’s not possible to be off by that much. That’s like one of China’s largest provinces not being there,” he commented. (The Guardian, 24 May 2017)

### Europe

➢ Several countries in Europe, including Switzerland, the Netherlands, Germany, Denmark and Spain, provide “fixing rooms” where users of hard drugs can take drugs under medical supervision, and with clean equipment. Although there are concerns that these facilities could encourage illegal drug use, the managers believe that they prevent drug users from publicly consuming drugs, and prevent fatal drug overdoses. The Skyen facility in Denmark provides harm reduction services, and will support users in cutting down or stopping their drug consumption, if desired. Other countries are following this initiative, with a hospital in Paris housing France’s first “fixing room”, and Glasgow is planning to open the UK’s first “fixing room”, looking to countries such as Denmark for inspiration. (BBC, 10 Jan 2016)

➢ The UK’s health secretary, Jeremy Hunt, confirmed that he did not expect the UK to remain within the European Medicines’ Agency (EMA) when the UK leaves the EU (“Brexit”). The former UK pharmaceutical regulator, Sir Alasdair Breckenridge, has warned this may led to delays in UK patients getting new drugs, including cancer drugs. The EMA authorises drugs for use across the EU, and companies may be slower in seeking permission in the UK alone if they need to pay for a separate assessment for their product, and the UK market is small compared to the EU. Jeremy Hunt is “hopeful” that the UK can work closely with the EMA, but David Jeffreys, the vice–president of Eisai – a Japanese pharmaceutical company – warns that UK patients could face delays of up to 2 years as the early innovations system will apply first in USA, Japan and the EU. He calls for a co–operation deal with the EMA, although there are risks that the issue may be absorbed into a wider debate on trade terms. A spokeswoman for the UK’s Department of Health said that ensuring the timely access to safe and effective medicines is a priority, and that Brexit is an opportunity to give even faster access to pharmaceutical innovations. (BBC, 10 February 2017)

➢ With the death of Prof Hans Rosling of the Karolinska Institute and co–founder of Gapminder, the world has lost a unique statistician, global health champion and communicator. The best way to remember Hans Rosling and champion his legacy is to keep push his key messages. His first message is that the world is evolving, and so should our perceptions on countries’ places on development pathways. We should ask more questions, and look for answers in evidence–based facts. Second, knowing the past makes it easier to believe in the future, as we grasp humanity’s progress in reducing poverty. Third, the rise of populism makes it even more vital to be vigilant and hold politicians accountable for the factual basis for their policy–making. Fourthly, we must share and apply scientific knowledge, especially in new and engaging ways, to make changes. And lastly, we must continue to “close the gap” between the message of statistics and our perceptions of the world. (Huffington Post, 17 February 2017)

➢ The Netherlands Minister for Foreign Trade and Development Co–operation launched the “She Decides” fund for family planning, to counter the US government’s “global gag rule”, which prevents non–US NGOs from providing services or information which relate to abortion (including counselling and legal advice), from receiving US government funding for any of their activities. Countries including Sweden, Belgium, Canada, the Netherlands, Finland, Denmark, Australia, Norway and Luxembourg have pledged more than US$ 110 million to the fund, with an additional US$ 20 million from the Bill and Melinda Gates Foundation, and US$ 50 million from an anonymous donor and US$ 10 million from Sir Christopher Hohn. The US application of the “global gag rule” could result in the loss of at least US$ 600 million of US funding, and some agencies believe its overall impact could be US$ 9.5 billion on issues such as HIV, maternal health and vaccinations. According to Alexander De Croo, Belgium’s Minister of Development Co–operation, the Sustainable Development Goals could not be achieved without sufficient access to family planning. “We cannot accept that the purely ideological decision of one country … would push millions of women and girls back into the dark ages. We will lead with our actions,” he said. (Devex, 2 March 2017)

➢ Nestlé will remove 10% of sugar from its confectionery products sold in the UK and Republic of Ireland – equivalent to 7500 tonnes each year. The move, following the possibility of a tax on sugary drinks, will lead to Nestlé replacing sugar with higher quantities of existing ingredients, and/or reducing product sizes. It may also involve the company’s touted “scientific breakthrough”, in which it claims to found a way to re–structure sugar, enabling it to reduce sugar content by up to 40%. Public Health England welcomed the announcement. “Nestlé is the latest household name to commit to making everyday products healthier, and we’re delighted that this is just the start of its efforts. This sends a clear message that reducing sugar in food is possible, even in products that are typically harder to reformulate,” says Duncan Selbie, the chief executive of Public Health England. (The Guardan, 7 March 2017)

### India

➢ According to the National Family Health Survey, the lack of quality neonatal care, especially in rural areas, is the main reason behind India’s high neonatal mortality rate. The neonatal mortality rate in urban areas is 29 per 1000 live births, compared to 41 per 1000 live births in rural areas – a total of 650 000 deaths each year. The main causes of death are premature births, infections and asphyxia, and many premature babies who survive suffer from disabilities including cerebral palsy, learning disabilities and respiratory conditions, resulting in physical, psychological and economic stress to the individual and their families. There is a severe shortage of neonatal intensive care units across India, Bhupendra Avasthi of the Surya Mother and Child Hospital, calls for public–private partnerships to set up more neonatal intensive care units in in rural and urban areas. (Times of India, 27 March 2017)

➢ Attempted suicide and “any act towards the commission” of suicide were repealed as criminal acts by India’s government in March 2017. These measures are part of a wider package of mental health reforms, which declares psychiatric care to be a right for all Indian people, along with increased funding. Before the repeal, people who attempted suicide faced a fine and imprisonment rather than support, while their families often had to pay bribes to avoid prosecution; and officials used the attempted suicide laws to lock up protesters who staged hunger strikes. India’s suicide rate is almost double that of the USA’s, and it lacks a suicide prevention plan; and measures such as limiting access to poison and tackling the taboos which prevent depressed people from opening up to friends and doctors could help. However, the main challenge lies in improving the lives of young people, where suicide is the leading cause of death, and the suicide rate for women aged 15–29 is more than double that of any other country except Surename. (Economist, 30 March 2017)

➢ The WHO confirmed that India has eliminated visceral leishmaniasis (also known as kala azar) in 82% of its sub–districts in 2015. The WHO also noted that Bangladesh achieved elimination in 97% of its sub–districts, and 100% of Nepal’s sub–districts achieved elimination. The importance of protection against financial risk was also highlighted by the WHO, as 25–75% of households in India, Bangladesh, Nepal and Sudan where some–one is affected by visceral leishmaniasis face some form of financial difficulties in obtaining diagnosis and treatment, even if diagnosis is free. Yaws were also eliminated in India, and the discontinuation of India’s Mass Drug Administration programme for lymphatic filariasis must be carefully monitored, while ensuring that de–worming coverage is maintained. The WHO recognizes that India has made significant progress against lymphatic filariasis in 72 sub–districts. India is among the countries with the highest prevalence of neglected tropical diseases (others include Brazil, China, Indonesia and Nigeria), and that 960 million of the 1.59 billion people requiring treatment for these diseases live in lower–middle rather than low–income countries. (Live Mint, 20 April 2017)

➢ In an attempt to reduce alcohol–related road accidents, India has banned the sale of alcohol within 500m of national and state highlights – a decision that affects shops selling alcohol, bars, restaurants and hotels. Binge drinking, with its resultant social and health problems are a large concern in India, where male drinkers aged over 15 years drank an average of 32.1 L of pure alcohol in 2010, 77% higher than the USA. Road deaths in India are also high, with 146 133 people killed in 2015. Many states ban alcohol entirely, while others are considering the move. The increasing restrictions on alcohol consumption have raised concerns among the drinks industry, who have been investing heavily in India, thanks to its large middle class and its status as the world’s largest whisky market by volume. The ban on sales near highways could severely affect tourist areas, eg, 85% of Goa’s alcohol shops could close or be forced to move. However, some states and shops are taking steps to circumvent the ban: states which rely on alcohol tax revenues have moved to reclassify highways as roads; and there are reports of shops moving their entrances to force people to travel more than 500m from the highway to enter them. (Wall Street Journal, 24 April 2017)

➢ According to the latest Global Burden of Disease study, published in *The Lancet*, India continues to perform poorly, ranking at 154 in the study’s index of the quality and accessibility of health care, behind countries such as China, Sri Lanka and Bangladesh. Despite India’s economic growth, it is failing to meet its health care goals and the gap between its actual and predicted score has widened over the past 25 years. Despite some gains, India performed worse than expected in tuberculosis, diabetes, rheumatic heart disease and chronic kidney disease. More broadly, the study also highlights inequalities in health care access and quality among countries at similar development levels, with China’s score of 74, ranking ahead of India’s score of 44.8, Sri Lanka scores 73, and Brazil and Bangladesh score 65 and 52 respectively. However, India is slightly ahead of Pakistan, at 43. (Times of India, 19 May 2017)

### The Americas

➢ Mayors across the USA have written to Congress on the effects of repealing the Affordable Care Act (ACA, or “Obamacare”), noting that it will be mainly felt at local level. The letter gathered more than 130 bi–partisan signatures, and calls for key provisions within ACA to be retained. These provisions include: insuring children up to the age of 26 years; eliminating lifetime and annual limits; assuring eligibility for insurance coverage even with pre–existing conditions; guaranteeing coverage for pregnancy and breast cancer screening; and providing coverage for preventive services at no additional cost. Martin Welsh, the Mayor of Boston and Chair of the US Conferences of Mayors Health Standing Committee, states that “health is not a privilege, it is a human rights. I am proud that mayors across the country are standing up in a bipartisan effort to improve the ACA, not repeal it.” (Cities Today, 24 February 2017)

➢ Hot tropical air is getting trapped in the narrow concrete alleys in many Latin American cities, causing stifling conditions and potential health problems. It increases the risk of mosquito–borne diseases, heat exhaustion, stress, and respiratory and cardiovascular conditions – and people suffering from conditions such as heart disease or hypertension are more prone to related complications. Poorer people are at increased risk from these heatwaves, as they may have inadequate water supplies or be further from hospitals. As well as the health impact, stifling air can worsen pollution, boost energy consumption and potentially curb economic activity. The problem of temperate spikes is caused by haphazard urban development, and the heat generated by cars, factories and buildings leading to “urban heat islands”. Temperature differentials are greater at night – when stagnant warm air becomes trapped – and may only be a few °C in some areas, but can differ by up to 20°C in others. Initially, urban heat islands were mainly a problem of megacities such as Mexico City and Rio de Janerio, but are rapidly spreading to other cities such as Santiago, Lima and Buenos Aires, and a 2015 study shows an increasing incidence of urban heat islands, coupled with a decline in city winds – and urban temperatures are rising more quickly than global temperatures. Changing building design and materials, improving airflows and planting trees may provide some relief to cities and their inhabitants, and in the longer–term changing the positioning of buildings, providing more green space, and promoting public transport over cars will decrease heat generation and improve air flow. (Reuters, 15 February 2017)

➢ The WHO confirmed that trachoma has been eliminated as public health problem in Mexico, making it the first country in the Americas (and the third globally, after Morocco and Oman) to eliminate the disease. Trachoma is transmitted by contact with eye bacteria and nasal discharges of infected people, and globally it is the main infectious cause of blindness. In Mexico, trachoma was endemic in 246 communities in the state of Chiapas, affecting over 146 000 people. Action to combat the disease were strengthened in 2004 with the creation of the Trachoma Prevention and Control Program, and strengthening the WHO SAFE strategy (a package of interventions including surgery for advanced cases, antibiotics, and hygiene and environmental improvement measures). Groups of doctors, nurses and technicians worked locally to reduce the number of cases from 1794 in 2004 to none in 2016, allowing Mexico to meet the criteria for elimination of trachoma as a public health threat. To prevent resurgence and maintain elimination, PAHO/WHO recommends continuous monitoring and care delivery to affected patients. (Outbreak News, 24 April 2017)

➢ With medicine shortages and escalating rates of malnutrition, Venezuela’s child mortality rate rose by 30% in 2016, and more than 11 000 babies died. The head of the country’s Ministry of Health was fired after these statistics were published. According to the Catholic aid agency, Caritas, child hunger is a “humanitarian crisis” in parts of Venezuela, with 11.4% of children aged under 5 suffering from severe or acute malnutrition, and 48% “at risk” of going hungry. A recent survey of 6500 families found that 75% of adults lost an average of 19 lb [8.6 kg] in 2016 – highly unusual outside of a war zone or an area hit by natural disasters. This crisis is largely man–made; with cash shortages and increasing debts, the government has cut back sharply on food imports. Despite ample fertile land, sunshine, water and cheap fuel, Venezuela’s farmers are unable to make up the shortfall, due to the country’s highly–inefficient system of agricultural production, with the government acting as producer, processor and distributor. Farmers lack sufficient hard currency to buy imported feed, fertilizers and spare parts, and the domestic production of rice, corn and coffee have fallen by at least 60% in the past decade. Venezuela’s currency, the bolivar, has lost 99% of its value in the past 5 years, meaning that a bag of rice worth US$ 1 sells for 6000 bolivars – about a day’s wages for the average worker. Farmers are only able to stay in business by breaking the law and selling at market prices, and also face extortions from criminal gangs, demanding money for “protection”. (Washington Post, 22 May 2017)

➢ According to a report by Save the Children, all 10 countries with the highest child homicide rates are in Latin America and the Caribbean, with Honduras having the highest rate. This is fostered by increases in violent criminal activity across the region, which also jeopardises children’s schooling and future prospects. Save the Children ranked countries based on indicators on child health, education, labour, marriage, childbirth and violence. The USA ranked relatively poorly, due to wide disparities between states on high–school dropout rates, food insecurity, rates of teenage pregnancies – and in 2015, 5000 children were murdered or committed suicide. “Children have the right to survival, food and nutrition, health and shelter. They also have the right to be encouraged and educated, both formally and informally. And they have the right to live free from fear, safe from violence and protected from abuse and exploitation,” said Save the Children. (NBC News, 31 May 2017)

